# Periostin facilitates ovarian cancer recurrence by enhancing cancer stemness

**DOI:** 10.1038/s41598-023-48485-8

**Published:** 2023-12-04

**Authors:** Zhiqing Huang, Olivia Byrd, Sarah Tan, Katrina Hu, Bailey Knight, Gaomong Lo, Lila Taylor, Yuan Wu, Andrew Berchuck, Susan K. Murphy

**Affiliations:** 1 Division of Reproductive Sciences, Department of Obstetrics and Gynecology, Duke University School of Medicine, Durham, USA; 2 Biostatistics & Bioinformatics, Division of Biostatistics, Biostatistics & Bioinformatics, Duke University, Durham, USA; 3 Division of Gynecologic Oncology, Department of Obstetrics and Gynecology, Duke University School of Medicine, Durham, USA; 4 Department of Obstetrics and Gynecology, Duke University Medical Center, 701 West Main Street, Suite 510, Duke, PO Box 90534, Durham, NC 27701 USA

**Keywords:** Cancer, Cell biology, Molecular biology, Medical research, Oncology

## Abstract

The lethality of epithelial ovarian cancer (OC) is largely due to a high rate of recurrence and development of chemoresistance, which requires synergy between cancer cells and the tumor microenvironment (TME) and is thought to involve cancer stem cells. Our analysis of gene expression microarray data from paired primary and recurrent OC tissues revealed significantly elevated expression of the gene encoding periostin (*POSTN*) in recurrent OC compared to matched primary tumors (*p* = 0.015). Secreted POSTN plays a role in the extracellular matrix, facilitating epithelial cell migration and tissue regeneration. We therefore examined how elevated extracellular POSTN, as we found is present in recurrent OC, impacts OC cell functions and phenotypes, including stemness. OC cells cultured with conditioned media with high levels of periostin (CM^*POSTNhigh*^) exhibited faster migration (*p* = 0.0044), enhanced invasiveness (*p* = 0.006), increased chemoresistance (*p* < 0.05), and decreased apoptosis as compared to the same cells cultured with control medium (CM^*CTL*^). Further, CM^*POSTNhigh*^-cultured OC cells exhibited an elevated stem cell side population (*p* = 0.027) along with increased expression of cancer stem cell marker CD133 relative to CM^*CTL*^-cultured cells. *POSTN*-transfected 3T3-L1 cells that were used to generate CM^*POSTNhigh*^ had visibly enhanced intracellular and extracellular lipids, which was also linked to increased OC cell expression of fatty acid synthetase (*FASN*) that functions as a central regulator of lipid metabolism and plays a critical role in the growth and survival of tumors. Additionally, *POSTN* functions in the TME were linked to AKT pathway activities. The mean tumor volume in mice injected with CM^*POSTNhigh*^-cultured OC cells was larger than that in mice injected with CM^*CTL*^-cultured OC cells (*p* = 0.0023). Taken together, these results show that elevated POSTN in the extracellular environment leads to more aggressive OC cell behavior and an increase in cancer stemness, suggesting that increased levels of stromal POSTN during OC recurrence contribute to more rapid disease progression and may be a novel therapeutic target. Furthermore, they also demonstrate the utility of having matched primary-recurrent OC tissues for analysis and support the need for better understanding of the molecular changes that occur with OC recurrence to develop ways to undermine those processes.

## Introduction

In the United States during the year 2023, there will be approximately 19,710 new cases of ovarian cancer (OC) and 13,270 deaths due to this disease^1^. OC is the second-most-common gynecologic malignancy, accounting for more deaths than any other cancer of the female reproductive system^2^. The main reasons for the higher death rate associated with OC are that it often goes undetected or misdiagnosed due to a lack of specific symptoms, the frequency of OC recurrence, and intrinsic or acquired chemoresistance. Treatment strategies have not significantly improved in the past 30 years, and immunotherapy is not effective with most OC patients. Currently, most patients receive neoadjuvant chemotherapy followed by interval debulking and then finish chemotherapy^3^. About half of the patients receive maintenance treatment with PARP inhibitors^4^. Yet 70–75% of individuals diagnosed with advanced stage serous OC will experience recurrent, incurable disease despite an initial promising response to treatment^5–7^. Although the genetics of primary OC have been extensively studied, little data is available on recurrent tumors. It is difficult to obtain recurrent tumor samples and even more difficult to obtain primary (pOC)—recurrent (rOC) tumor pairs^7^. Investigation into the genomic differences that characterize rOC, and how these differences contribute to tumor progression and chemotherapeutic response, is of great importance in the effort to identify relevant targets that allow for more effective treatment of patients with OC.

Tumor heterogeneity and the need to minimize toxicity to normal cells presents a tremendous challenge for treating chemoresistant disease^8^. The tumor microenvironment (TME) plays an important role in the development of acquired chemoresistance^9,10^. The TME includes the blood vessels, immune cells, fibroblasts, signaling molecules, and extracellular matrix (ECM) that surround a tumor^11,12^. The ECM is perturbed in tumors and can promote the growth, survival, and invasion of cancer; the ECM also modifies fibroblast and immune cell behavior to promote metastasis and impair response to treatment^13^.

The ECM is a complex assembly of fibrous proteins, proteoglycans, and other molecules, including cytokines and growth factors^14^. Matricellular proteins play a central role in the homeostasis of normal tissues regulating cell proliferation and differentiation^14,15^. These proteins are generally expressed at low levels in most adult tissues but are highly expressed during inflammation, tissue repair, wound healing, and malignant transformation^15^. Our analysis of gene expression microarray data from paired primary and recurrent OC tissues revealed significantly elevated expression of multiple ECM genes in recurrent OC compared to matched primary tumors. Among these, periostin, encoded by the gene *POSTN*, is a key player in tissue repair and remodeling and plays a role in several immune-mediated inflammatory conditions and in cancer development and progression^16^. Periostin is a secreted extracellular matrix protein that is required for maintaining the cell microenvironment during normal cell growth and proliferation. Periostin binds to integrins to support adhesion and migration of epithelial cells^17^. Elevated expression of *POSTN* and of the periostin protein have been reported in cancer cells and in the TME^16^ and are associated with poor prognosis as well as resistance to chemotherapeutic treatments, including in OC^18^.

Periostin is known to be involved in the promotion of cancer cell growth, cancer invasion, and chemoresistance^16^, but the role of periostin in the TME, especially its contribution to cancer recurrence, has not been fully investigated. Here we used in vitro and in vivo models to examine the role of exogenous periostin in ovarian cancer cell phenotypes, including growth, stemness, and chemosensitivity.

## Results

### Periostin is highly expressed in recurrent ovarian cancer

**Figure 1:**
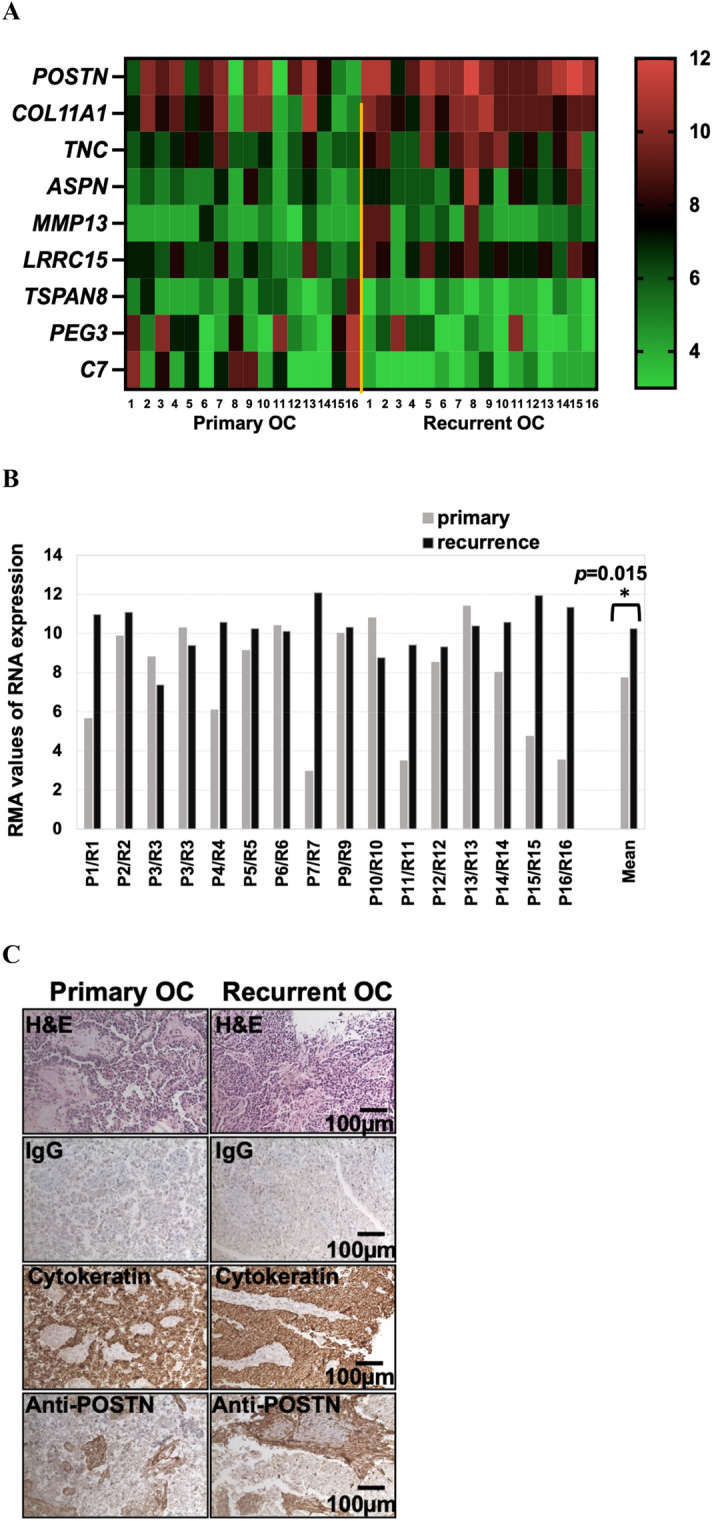
Periostin is highly expressed in recurrent ovarian cancer. (**A**) Gene expression microarray results for sixteen paired pOC and rOC tissues. The heatmap shows data from nine genes with > twofold differences in expression. (**B**) *POSTN* expression in each matched pOC–rOC pair. *POSTN* is increased in the rOC specimens for 11 of the 16 pairs (*p* = 0.015). Microarray assay was performed once per sample. (**C**) IHC staining using an anti-periostin antibody shows elevated periostin protein in rOC relative to pOC, with higher levels present in the stroma. Micrographs were taken at a total magnification of × 100.

To investigate gene expression changes in tumors that distinguish recurrent from primary ovarian cancers, we generated gene expression microarray data using Affymetrix U133 Plus 2.0 arrays for 16 matched primary (pOC) and recurrent (rOC) high grade serous epithelial ovarian cancer tissues^19,20^ (Supplementary Table 1). All tumor pairs were classified as having papillary serous histology by pathological exam. There were 642 genes showing significant differential expression between pOCs and rOCs (*p* < 0.05). Among these genes, nine exhibited a greater than two-fold difference in expression (Fig. 1A). Six of these had more than two-fold higher expression in rOC, including periostin (*POSTN*), collagen type XI alpha 1 chain (*COL11A1*), tenascin C (*TNC*), asporin (*ASPN*), matrix metalloproteinases 13 (*MMP13*), and leucine rich repeat containing 15 (*LRRC15*). Three exhibited more than two-fold lower expression in rOC, including complement C7 (*C7*), paternally expressed gene 3 (*PEG3*), and tetraspanin 8 (*TSPAN8*). Interestingly, all six genes with elevated expression in rOC vs pOC belong to the extracellular matrix (ECM) family. Of these, *POSTN* showed the most marked difference in expression between the groups, with 11 of the 16 pairs exhibiting higher expression in rOC (*p* = 0.015) (Fig. 1B). To confirm that potential heterogeneity in the stromal versus tumor content of the individual specimens was not contributing to this difference, we re-analyzed these data after normalizing *POSTN* expression to the expression of genes identified as being specific to either the ovarian cancer stroma (*MTSS1*^21^) or to normal stroma tissue (*RAPGEF2*^21^). Both *MTSS1-*normalized and *RAPGEF2*-normalized *POSTN* levels retained significant differences between the pOC and rOC (Supplementary Fig. 1, *p* = 0.008 and *p* = 0.006, respectively). Immunohistochemistry staining confirmed that periostin protein levels were elevated in rOC relative to the matched pOC and exhibited higher levels in the stromal tissues than in the cancer cells (Fig. 1C). The images were analyzed using Image J (Supplementary Table 2), which showed a larger signal area and a higher intensity of staining for periostin in the rOC versus the pOC (signal area: 34.5% vs 7.9%; and mean signal values: 87.9 vs 20.2 pixels, respectively).

### Extracellular periostin enhances invasiveness and chemoresistance in vitro

**Figure 2:**
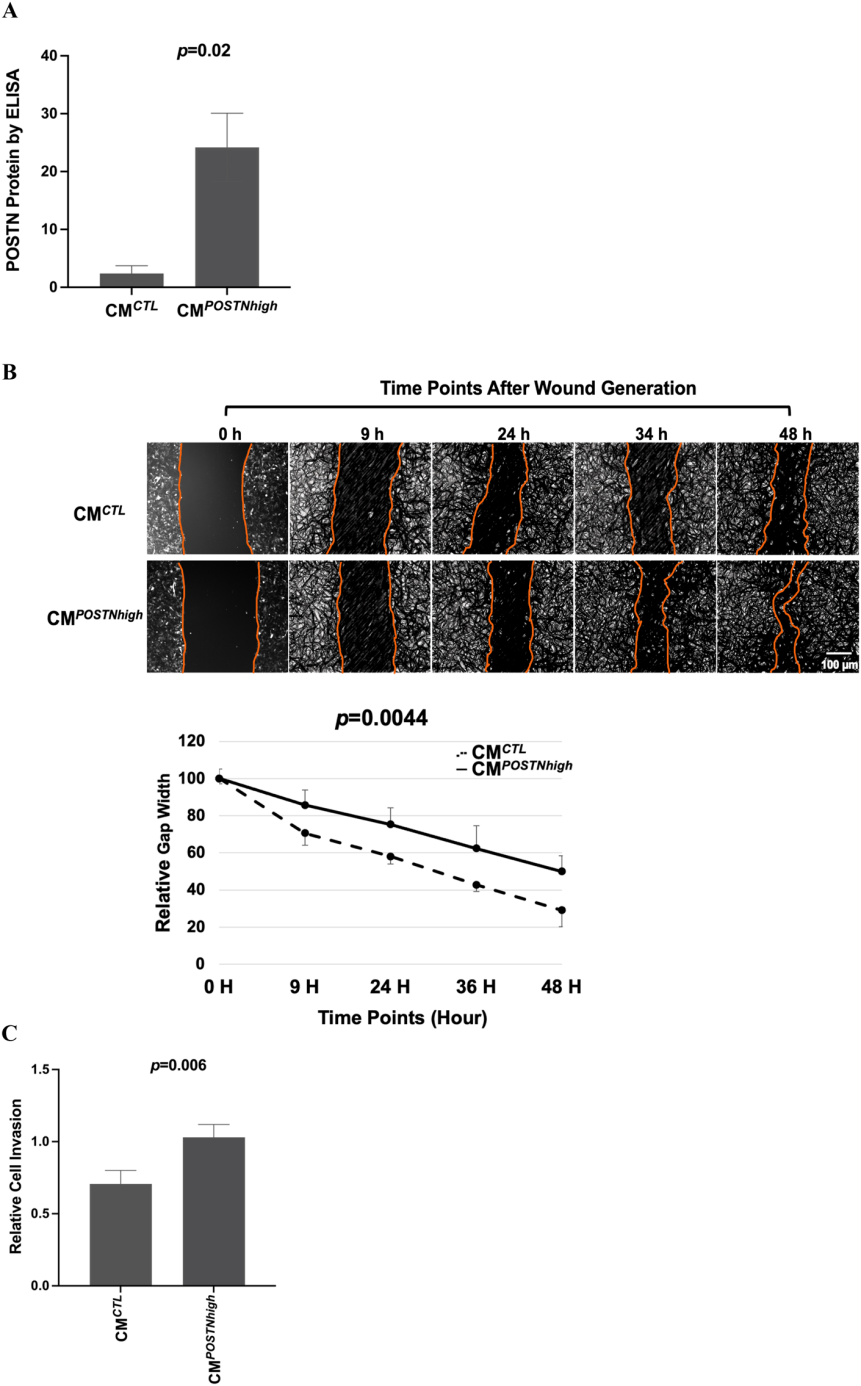

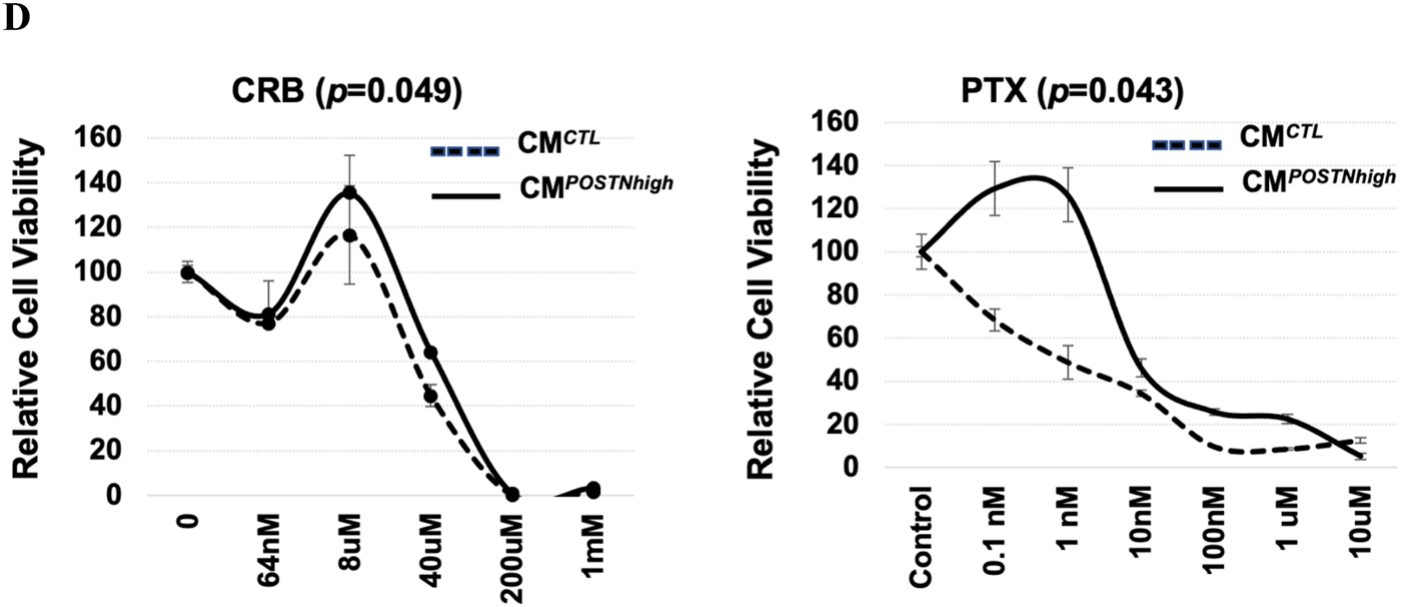
Higher periostin expression in the tumor cell environment leads to more aggressive cancer phenotypes. (**A**) ELISA assay results showing periostin protein levels in the conditioned medium from 3T3-*POSTN* cells (CM^*POSTNhigh*^) compared to 3T3-CTL cells (CM^*CTL*^), normalized to the total protein level (*p* = 0.02). This test was repeated independently two times with three replicates of 3T3-CTL and 3T3-*POSTN* samples each test. (**B**) HEYA8 cells exhibit faster migration and proliferation (‘wound healing’) under CM^*POSTNhigh*^ conditions compared to CM^*CTL*^ conditions. (Scale bar, 100 µm; × 4 magnification). Opposing cell fronts defining the wound gaps are indicated. The mean gap widths measured from four arbitrary points at each time point are plotted against time for each culture condition in the graph below. (**C**) HEYA8 cells are more invasive when cultured under CM^*POSTNhigh*^ vs CM^*CTL*^ (*p* = 0.006). Results of three independent experiments shown. (**D**) Culture under CM^*POSTNhigh*^ vs CM^*CTL*^ promotes chemoresistance in A2780 cells (*p* = 0.049 for carboplatin [CRB] and 0.043 for paclitaxel [PTX]. Results shown from three independent experiments, each performed in sextuplicate.

Prior reports^22–24^ suggested that cancer stromal tissues express high levels of periostin and that this is related to poorer prognosis. However, the effects of elevated exogenous levels of periostin in cancers, such as those in the tissue microenvironment, have not been extensively investigated. To this end, we generated a stable mouse 3T3-L1 preadipocyte cell line with plasmid pLenti-GIII-CMV-RFP-2A-Puro-*POSTN* that results in high levels of periostin protein expression and used this alongside a control plasmid (pLenti-CMV-RFP-2A-Puro-Blank Vector). Due to the co-expression of red fluorescent protein (RFP), we were able to select *POSTN* + cells using fluorescence-activated cell sorting with the RFP marker. RT-PCR with a *POSTN* probe showed significantly higher expression of *POSTN* in 3T3-*POSTN* cells (Supplementary Fig. 2, *p* = 0.003). In Fig. 2A, ELISA was performed using the medium collected from either *POSTN*-transfected 3T3-L1 cells (3T3-*POSTN*) or vector-transfected cells (3T3-Blank) following at least 48 h in cell culture. The conditioned medium (CM) from 3T3-*POSTN* cells showed a significant induction of periostin protein (CM^*POSTNhigh*^) compared to CM from 3T3-blank cells (CM^*CTL*^, *p* = 0.02).

To determine the impact of *POSTN* on cell migration, the ovarian cancer cell line HEYA8 was cultured in media containing CM^*POSTNhigh*^ or CM^*CTL*^ and assessed with a wound healing assay. As shown in Fig. 2B, HEYA8 cells cultured with CM^*POSTNhigh*^ exhibited faster cell migration, evidenced by the shorter time taken to close the gap that was introduced in the cell monolayer. The difference in gap closure time between CM^*POSTNhigh*^ and CM^*CTL*^ was statistically significant (*p* = 0.004). We next tested the influence of periostin on cell invasion. HEYA8 cells were added to the top chamber of the Boyden chamber invasion assay and were allowed to migrate through pores to the opposite side of the membranes into either CM^*CTL*^ or CM^*POSTNhigh*^, both of which contained 20% serum. Cells that had migrated through the membrane were then stained and counted. HEYA8 cells exhibited significantly more invasion into the CM^*POSTNhigh*^ than the CM^*CTL*^ environment, as shown in Fig. 2C (*p* = 0.006).

We next determined how *POSTN* influences response to carboplatin and paclitaxel, two commonly used chemotherapeutic agents for treatment of epithelial ovarian cancer. The OC cell lines HEYA8 and CAOV2 are resistant to these drugs, so we instead used A2780 OC cells in this study. A2780 cells were cultured with either CM^*POSTNhigh*^ or CM^*CTL*^. The CM^*POSTNhigh*^ culture conditions enhanced chemoresistance of A2780 cells to both carboplatin and paclitaxel as compared to A2780 cells grown in CM^*CTL*^ conditions (Fig. 2D) (*p* = 0.049 and *p* = 0.043 for carboplatin and paclitaxel, respectively).

### Periostin is protective against apoptosis

**Figure 3:**
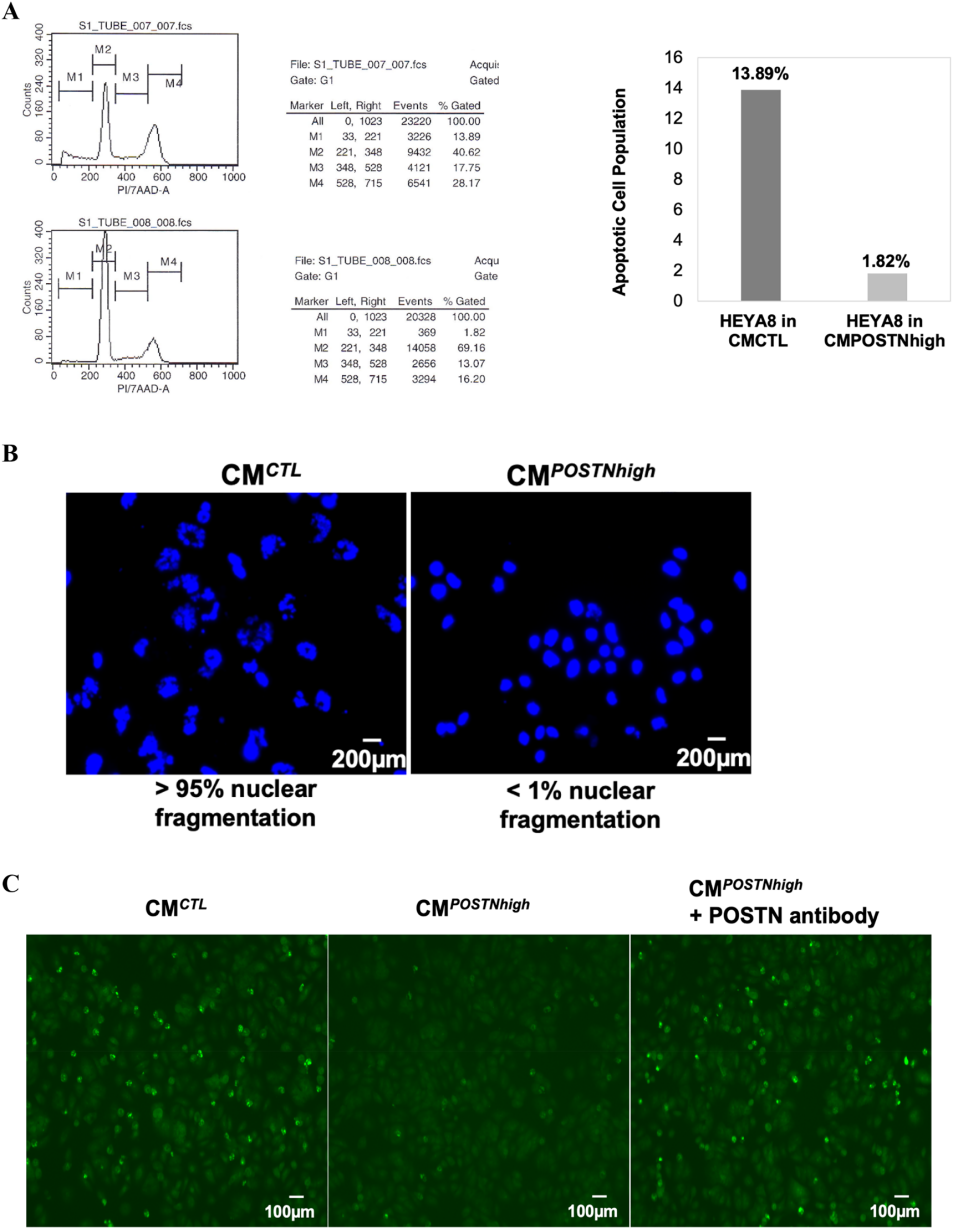
Increased resistance to apoptosis with high levels of periostin in the microenvironment. (**A**) HEYA8 cells resist apoptosis (M1 phase) induced by paclitaxel (PTX) when cultured under CM^*POSTNhigh*^ as compared to cells grown under CM^*CTL*^. This test was also performed in CAOV2 cells (Supplementary Fig. 3). The graph shows representative results from flow cytometry cell cycle analysis using HEYA8 cells. This test was independently repeated three times. (**B**) After treatment with paclitaxel, HEYA8 cell nuclei, stained with DAPI, exhibit less fragmentation when cultured under CM^*POSTNhigh*^ compared to CM^*CTL*^ conditions. The percentage of fragmented nuclei per field is shown under each image (20 × 10 magnification). This test was repeated twice. (**C**) Cell apoptosis was assessed by immunohistochemistry examination for caspase-3 levels. Caspase-3 activity was lower in HEYA8 cells when they were cultured with CM^*POSTNhigh*^ (middle panel) when compared to CM^*CTL*^ culture (left panel). Caspase-3 staining increased in HEYA8 cells cultured with CM^*POSTNhigh*^ when they were also treated with a neutralizing periostin antibody (right panel) (10 × 20 magnification). This test was repeated twice.

To investigate the influence of extracellular periostin on apoptosis of cancer cells, we performed propidium iodide (PI) staining and a flow cytometry assay with HEYA8 cancer cells cultured with elevated periostin levels (CM^*POSTNhigh*^). We found that the number of cells in the M1 phase (representing cell apoptosis, cell death, and cell debris) was reduced in CM^*POSTNhigh*^-cultured HEYA8 cells versus CM^*CTL*^-cultured cells when treated with paclitaxel (Fig. 3A; 1.82% in CM^*POSTNhigh*^ versus 13.89% in CM^*CTL*^). Similar results were observed for another ovarian cancer cell line, CAOV2, and using 1 μM staurosporine as the inducer of apoptosis (Supplementary Fig. 3A,B).

Nuclear fragmentation is a prominent morphological feature of cells undergoing apoptosis^25^, so we examined cell morphology following staining with DNA content dye Hoechst 33342. The nuclei showed less fragmentation in the HEYA8 cells cultured with CM^*POSTNhigh*^ (< 1%) compared to the cells cultured with CM^*CTL*^ (> 95%) (Fig. 3B), suggesting that extracellular periostin contributes to apoptotic resistance. Caspases are critical mediators of apoptosis, and caspase-3 is a death protease frequently activated during this process^26^. We therefore examined caspase-3 levels in HEYA8 cells cultured for 96 h with either CM^*CTL*^, CM^*POSTNhigh*^, or CM^*POSTNhigh*^ plus a monoclonal antibody to human periostin using the NucView® 488 caspase-3 assay kit (Fig. 3C). Apoptosis was induced using 100 nM of paclitaxel for 24 h. As shown in Fig. 3C, caspase-3 activity was lower in HEYA8 cells when they were cultured with CM^*POSTNhigh*^ (Fig. 3C middle panel) than when HEYA8 cells were cultured with CM^*CTL*^ (Fig. 3C left panel). Inclusion of the neutralizing periostin antibody with the CM^*POSTNhigh*^ resulted in increased apoptosis, evident from the increase in caspase-3 activation (Fig. 3C right panel), indicating that the apoptotic inhibition observed in cells cultured with CM^*POSTNhigh*^ was alleviated by the periostin-specific antibody. Supplementary Table 3 shows the average intensity (mean) and the percentage of pixels (% area) of caspase-3 staining measured by ImageJ, which confirms that staining intensity and percentage of pixels decreased in cells cultured with CM^*POSTNhigh*^ compared to the OC cells cultured with CM^*CTL*^ and that this reduced caspase-3 signal was partially reversed when the neutralizing *POSTN* antibody was added to the medium. These data indicate that apoptosis inhibition in HEYA8 cells is partially attributable to specific effects of periostin in the extracellular environment.

### POSTN-CM enhances the OC side population of stem-like cancer cells

**Figure 4:**
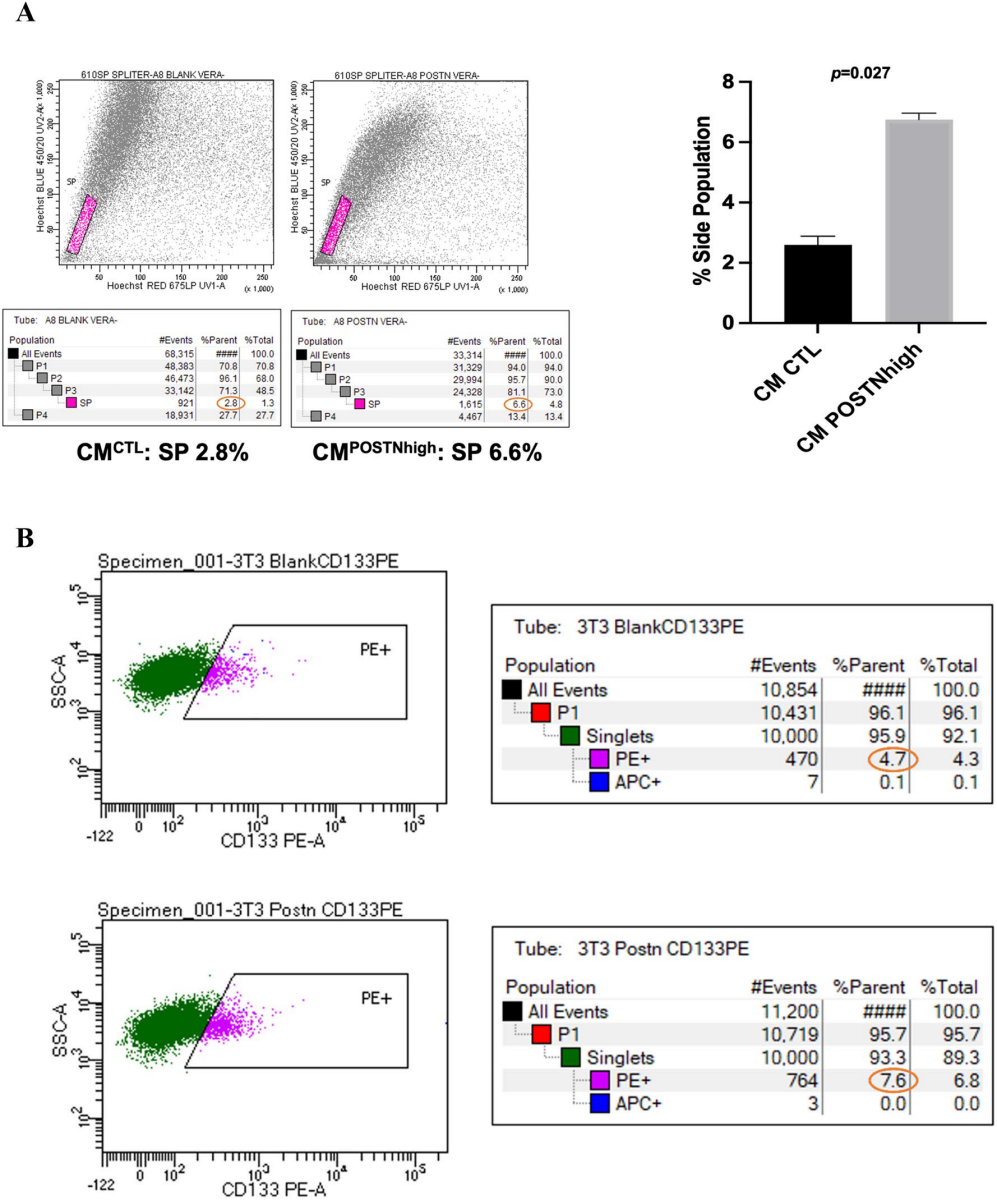
CM^*POSTNhigh*^ culture enhances cancer cell stemness. (**A**) The HEYA8 cancer stem cell-like side population (SP) is increased in cells grown under CM^*POSTNhigh*^ conditions when compared to cells grown under CM^*CTL*^ conditions following H33342 fluorescence staining and verapamil treatment as a control. The average side population percentage from duplicate measures of each CM condition is shown on right with a graph (*p* = 0.027). Representative flow cytometry data from HEYA8 cells for the side population analysis is shown on left. This test was independently performed twice. (**B**) The phycoerythrin-positive (CD133-positive) cell population is increased in CM^*POSTNhigh*^ conditions relative to control CAOV2 cells. Representative data from three experiments is shown.

Cancer stem cells have been shown in numerous cancer models to be involved in tumor development, cell proliferation, metastasis, and tumor recurrence due to their capacity for sustained self-renewal and genomic instability^27,28^. Several techniques have been developed to identify cancer stem cells including measurement, by FACS analysis, of the proportion of cells in a population that are able to efflux the Hoecsht dye H33342, referred to as a “side population”^29^. To determine the stemness of OC cells in the context of exogenous periostin, HEYA8 ovarian cancer cells were cultured in 3D culture conditions for 72 h with either CM^*CTL*^ or CM^*POSTNhigh*^ followed by evaluation using the side population assay. The mean percentage of side population cells from the cancer cells cultured in CM^*POSTNhigh*^ or CM^*CTL*^ was calculated from replicate tests. The cells cultured in CM^*POSTNhigh*^ exhibited a significantly larger side population as compared to the control cells (*p* = 0.027) (Fig. 4A). Similar results were obtained using the CAOV2 and SKOV3 OC cell lines (Supplementary Fig. 4). To support the findings from the side population analysis, we performed flow cytometry analysis for the cancer stem cell marker CD133^30^. As shown in Fig. 4B, the CD133-positive cell population was higher in CAOV2 cells cultured with CM^*POSTNhigh*^ (7.6%) than in CAOV2 cells cultured with CM^*CTL*^ (4.7%).

### Influence of periostin on lipid metabolism in the cancer microenvironment and in cancer cells

**Figure 5:**
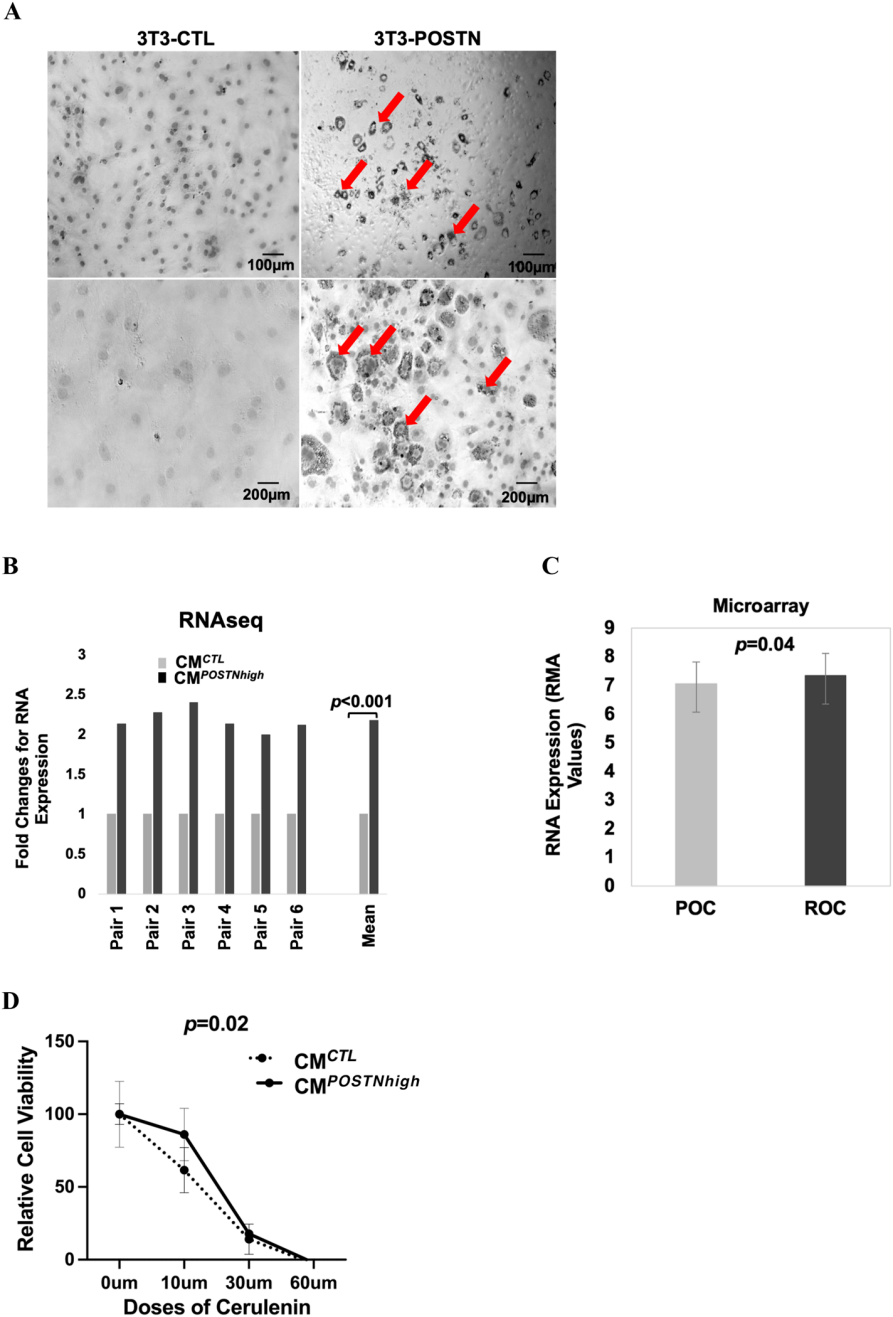

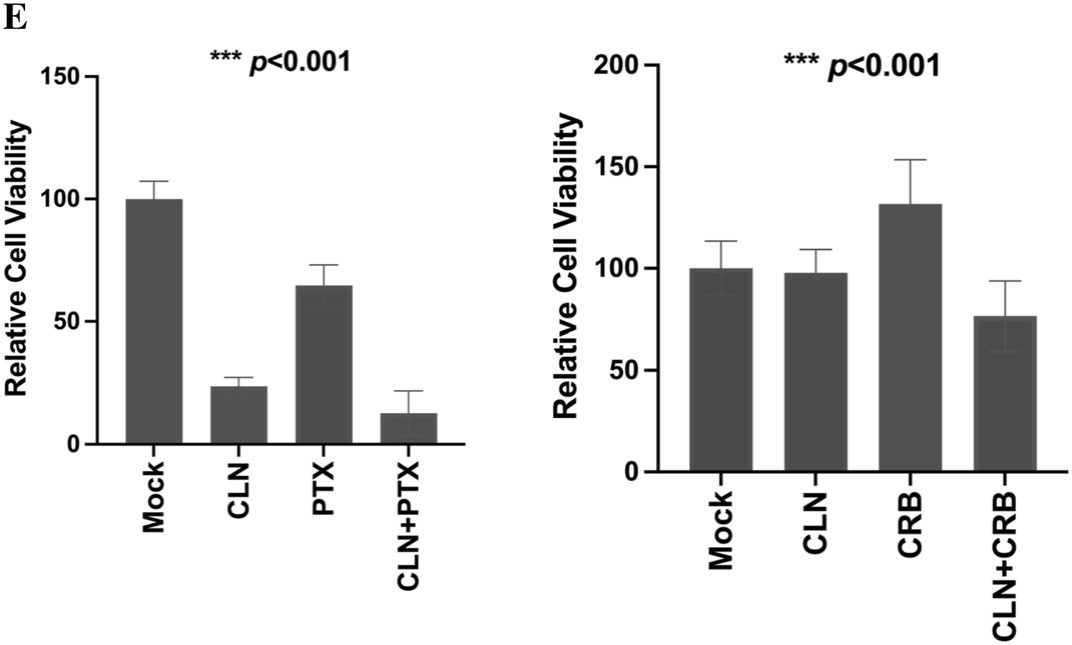
Exogenous periostin promotes lipid accumulation and is linked to lipid metabolism in the cancer microenvironment and in cancer cells. (**A**) *POSTN-*overexpressing 3T3-L1 preadipocytes exhibit enhanced lipid production. Imaging was performed in differentiated cells after Oil Red O staining at 10 × 10 magnification. The lipid drops stained with Oil Red O are indicated with an arrow. This test was independently repeated three times. (**B**) RNA sequencing data shows that the fatty acid synthesis gene (*FASN*) is more highly expressed in HEYA8 cells cultured under CM^*POSTNhigh*^ compared to CM^*CTL*^ (six replicates; *p* < 0.001). This test was independently repeated three times. (**C**) *FASN* expression is higher in rOC than in matched pOC (*p* = 0.04). This test was independently repeated three times. (**D**) HEYA8 cells grown under CM^*POSTNhigh*^ conditions are more resistant to FASN inhibitor Cerulenin (0–60 µM) than those grown with CM^*CTL*^ (*p* = 0.02). This test was independently repeated three times. (**E**) FASN inhibition combined with chemotherapy exhibits enhanced efficacy over that of either agent alone against HEYA8 cells under CM^*POSTNhigh*^. Left, cells treated with cerulenin (CLN), paclitaxel (PTX), or CLN + PTX; right, cells treated with CLN, carboplatin (CRB), or CLN + CRB. This test was independently repeated three times.

When *POSTN* was overexpressed in 3T3-L1 preadipocyte cells (3T3-*POSTN*) via transfection with a *POSTN* plasmid, we noticed that the cell morphology changed. The 3T3-*POSTN* cells were larger after cell differentiation was induced as compared to the 3T3-CTL cells. We then performed staining with Oil Red O, a fat-soluble dye that stains neutral triglycerides and lipids. The 3T3-*POSTN* cells exhibited a higher proportion of lipids in the cytoplasm than did the 3T3-CTL cells (Fig. 5A). This data supports the idea that those higher levels of periostin in preadipocyte cells are associated with an increased presence of lipids in the cytoplasm.

To explore the functional contribution of a lipid-enriched environment on cancer cell lipid metabolism, HEYA8 cells were cultured in 3D conditions with either CM^*CTL*^ or CM^*POSTNhigh*^ followed by transcriptome analysis by RNAseq (Fig. 5B). We found expression of Fatty Acid Synthase (*FASN*) was significantly increased in cancer cells when cultured using CM^*POSTNhigh*^ (*p* < 0.001). We also found that the expression of *FASN* in rOCs was significantly higher than in the matched pOC (*p* = 0.04) (Fig. 5C). Our data suggest that FASN, which is important for catalyzing fatty acid synthesis in cancer, is functionally responding to the elevated levels of periostin in the tumor microenvironment, and that the increased expression of FASN in cancer cells may lead to increased fatty acid synthesis.

To determine how exogenous periostin impacts FASN function, HEYA8 cells were grown in CM^*CTL*^ or CM^*POSTNhigh*^ and treated with the FASN inhibitor cerulenin (CLN). The cells showed increased sensitivity to the FASN inhibitor when cultured in CM^*CTL*^ than when cultured in CM^*POSTNhigh*^ (Fig. 5D, p = 0.02). These results indicate that, in the context of higher periostin in the microenvironment, OC cells are more resistant to the chemotherapeutic effects of this FASN inhibitor.

Cerulenin can enhance antitumor activity when combined with chemotherapy reagents in human colon cancers^31^. We therefore tested the effect of FASN inhibition on OC cells’ responses to paclitaxel. HEYA8 cells grown in CM^*POSTNhigh*^ medium were mock treated or treated with paclitaxel (PTX), cerulenin (CLN), or CLN + PTX (Fig. 5E, left panel). The data show that CLN and PTX combined reduced cell viability, and this effect was significantly stronger than the effect of either agent on its own (vs PTX only: *p* < 0.0001, 51.9% decrease; vs CLN: *p* = 0.01, 11% decrease). This indicates that FASN inhibition modulates the effects of taxane-based chemotherapy on OC cells. Similar results were also obtained with CLN and carboplatin (CRB) (Fig. 5E, right panel). When treated with the single agents, HEYA8 was resistant to CRB (*p* < 0.0001, 32% increase) and sensitive to cerulenin (*p* < 0.0001, 29% decrease). When combined (CLN + CRB), the chemoresistance observed with carboplatin alone (CRB) was partially reversed. These data suggest that when OC cells are exposed to exogenous periostin which is associated with increased lipids and chemoresistance, the inhibition of FASN activity can enhance chemotherapeutic effects.

### Periostin interacts with the AKT pathway

**Figure 6:**
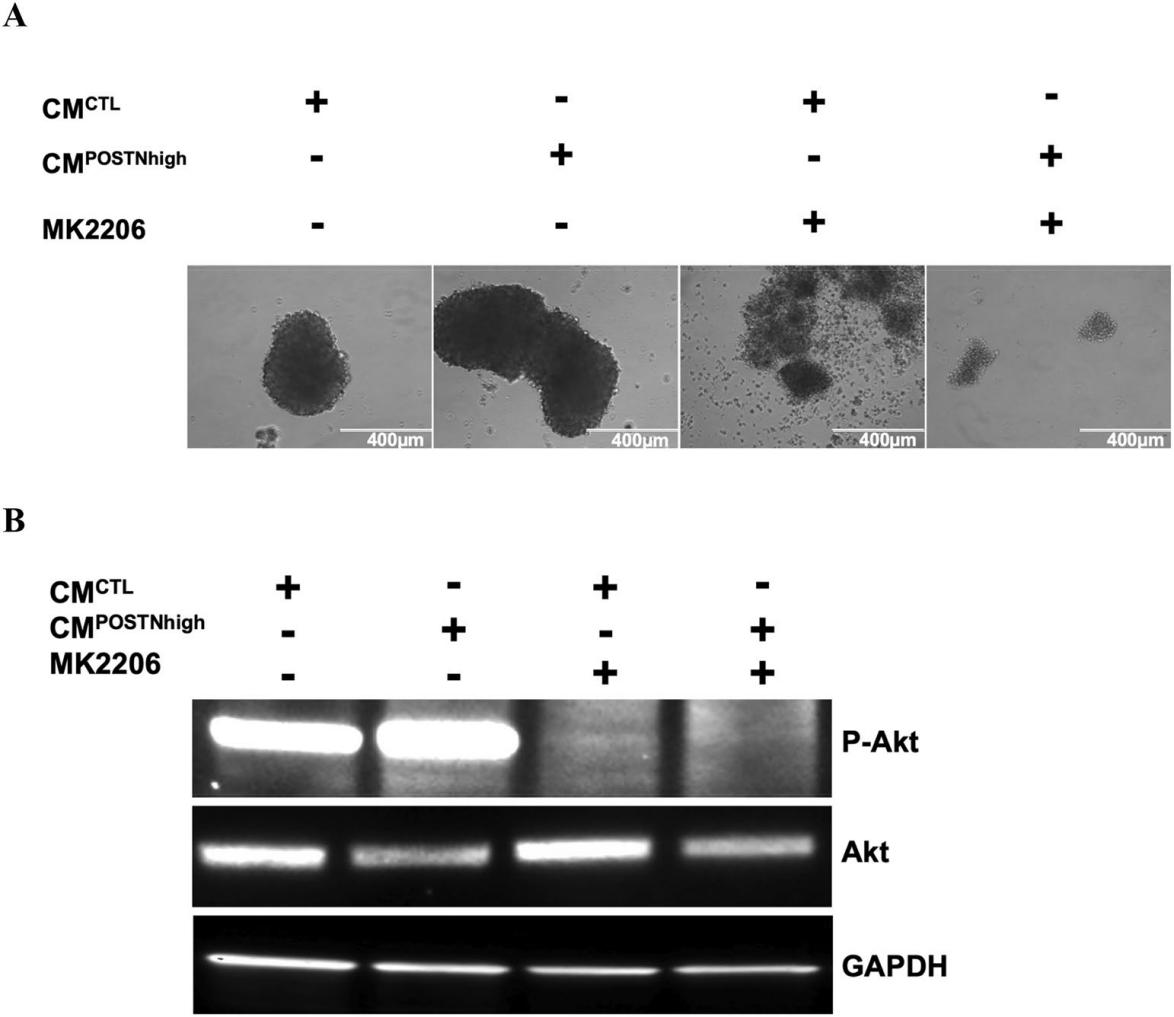
The AKT pathway contributes to *POSTN* regulation in the cancer microenvironment. (**A**) HEYA8 spheroid formation is disrupted under CM^*CTL*^ and CM^*POSTNhigh*^ conditions when cells are treated with AKT inhibitor MK-2206, with more substantial disruption when the cells are cultured in CM^*POSTNhigh*^. (**B**) AKT activation after treatment with AKT inhibitor MK-2206 is increased in HEYA8 cells cultured with CM^*POSTNhigh*^ compared to HEYA8 cells cultured with CM^*CTL*^. Western blots are shown using an anti-phospho-AKT antibody (top panel) or anti-total AKT antibody (middle panel). GAPDH was used as the internal loading control (bottom panel).

The PI3K/AKT/mTOR pathway is activated in approximately 70% of OCs and plays important roles in promoting cancer growth, proliferation, and cell survival through an intricate series of hyperactive signaling cascades^32^. To investigate the roles of the PI3K/AKT/mTOR pathway in cancer cells when exposed to a high-*POSTN* environment, we cultured HEYA8 cells in 3D with either CM^*POSTNhigh*^ or CM^*CTL*^ medium, CM^*CTL*^ + MK2206 (AKT inhibitor), or CM^*POSTNhigh*^ + MK2206 for 72 h. As shown in Fig. 6A, the cancer cells formed spheroids in both CM^*CTL*^ and CM^*POSTNhigh*^ culture conditions. Spheroids were visibly larger, denser and in increased numbers for HEYA8 cells in CM^*POSTNhigh*^ culture (Fig. 6A, Supplementary Fig. 6 and Supplementary Table 4). The spheroids were smaller and more disaggregated when cells were exposed to MK-2206 in CM^*CTL*^ medium. With MK-2206 present in CM^*POSTNhigh*^ medium, there were even fewer spheroids, and they were further disrupted. Western blotting demonstrated that phospho-AKT was present at higher levels in the HEYA8 cells cultured with CM^*POSTNhigh*^ compared to CM^*CTL*^-cultured cells (Fig. 6B). Phospho-AKT was inhibited by MK-2206 in both *POSTN*-low (CM^*CTL*^) and *POSTN*-high (CM^*POSTNhigh*^) culture conditions.

### High levels of periostin in the tumor environment enhance tumor formation in vivo

**Figure 7:**
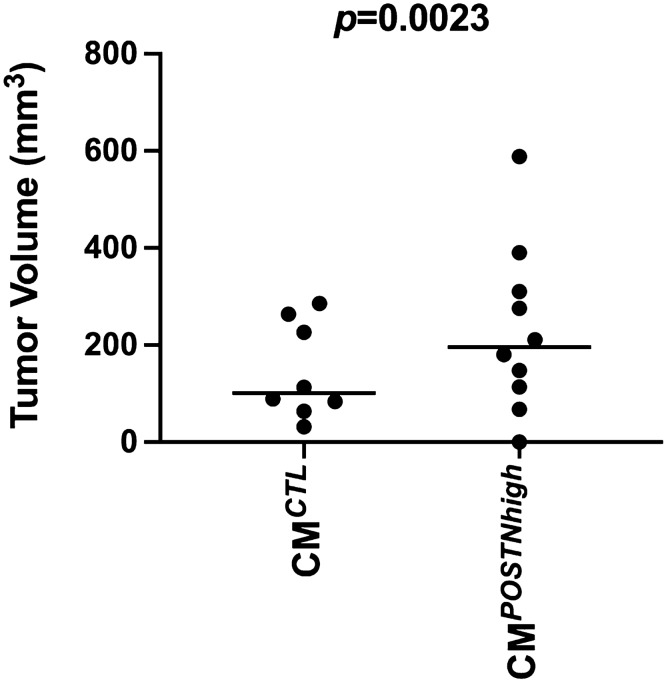
Ovarian cancer cells cultured with CM^*POSTNhigh*^ exhibit increased cancer formation in vivo. Female athymic nude mice were subcutaneously injected with 5 × 10^6^ CAOV2 cells suspended in CM^*POSTNhigh*^ and ECM Matrigel at a 1:1 ratio (n = 10). Mice in the CM^*CTL*^ group (n = 8) were injected with 5 × 10^6^ CAOV2 cells suspended in CM^*CTL*^ and ECM Matrigel at a 1:1 ratio. Tumors were measured and tumor volume calculated on Day 20 after removal. Mean tumor volume was higher in mice injected with cancer cells cultured in CM^*POSTNhigh*^ (mean = 228.4 mm^3^, 95% CI 105.7 to 351.2) compared with tumors from cancer cells cultured in CM^*CTL*^ (mean = 144.6 mm^3^, 95% CI 62.33 to 227.0) (*p* = 0.0023).

We used a xenograft mouse model of serous epithelial OC to determine if exogenous periostin contributes to tumor formation in vivo. Because CAOV2 cells were derived from a patient with high grade serous OC and this cell line has been used in our lab in many other studies^19,33,34^ including generation of OC xenograft mouse models, we used CAOV2 cells for this study. Two groups of female athymic nude mice were used, with 10 mice in the CM^*POSTNhigh*^ group and 8 mice in the CM^*CTL*^ group. On Day 0, the mice in the CM^*POSTNhigh*^ group were subcutaneously injected with CAOV2 cells (5 × 10^6^ per mouse) resuspended in CM^*POSTNhigh*^ medium and ECM Matrigel at a 1:1 ratio. Mice in the CM^*CTL*^ group were subcutaneously injected with the same number of CAOV2 cells resuspended in CM^*CTL*^ medium and ECM Matrigel at a 1:1 ratio. Tumor length and width were measured on Days 11 and 18 after cancer cell injection using a caliper (Supplementary Table 5). On Day 20, the mice were euthanized, and tumor length and width were measured again after tumor removal (Fig. 7). At Day 20, 9 out of 10 mice (90%) in the CM^*POSTNhigh*^ group and 8 out of 8 mice (100%) in the CM^*CTL*^ group showed tumor formation. Average tumor volume in the CM^*POSTNhigh*^ group was larger, although not significantly so, on Day 11 (mean of 21.3 mm^3^ in CM^*POSTNhigh*^ group vs 11.9 mm^3^ in CM^*CTL*^ group, *p* = 0.513*)* and Day 18 (mean of 170.2 mm^3^ in CM^*POSTNhigh*^ group vs 108.1 mm^3^ in CM^*CTL*^ group, *p* = 0.419). The difference was significant by Day 20 (228.4 mm^3^ in CM^*POSTNhigh*^ group vs 144.6 mm^3^ in CM^*CTL*^ group, *p* = 0.002; Fig. 7). During the 20-day study, the tumors showed local spread, without evidence of metastasis to other organs.

## Discussion

In the present study, we report that periostin is expressed in both stromal tissues and cancer cells and that expression is frequently increased in recurrent ovarian cancer as compared to the matched primary ovarian cancer from the same patient. We further found in vitro that OC cells cultured in the presence of higher exogenous periostin levels from conditioned media have a more aggressive phenotype, including increased proliferation and invasiveness, resistance to apoptosis, enhanced stemness, and enhanced chemoresistance. In vivo, OC cells cultured with higher exogenous periostin showed more aggressive tumor formation relative to control cells. Recent studies^35^ have shown that periostin expression has the potential to promote OC cell growth, migration, and invasion. Lozneanu et al.^36^ performed immunohistochemical studies of periostin using 102 samples of different histological OC subtypes. They reported that periostin is expressed in both cancer cells and stromal cells, and its expression in tumor cells and stroma associates with clinical features including age, histological type, tumor recurrence, and prognosis. In short, they found that higher stromal periostin levels were correlated with worse clinical features and poorer prognosis, while periostin expression in tumor cells seemed to be associated with better patient outcomes. Other studies^22–24^ have also demonstrated the clinical relevance of stromal periostin and its correlation with poor prognosis and worse clinical outcomes. Our results corroborate others’ earlier findings^22–24,35,36^ showing positive correlations between periostin expression in OC stromal tissues and advanced-stage OC.

We found that *POSTN* frequently exhibits higher expression in recurrent relative to matched primary ovarian cancers. As periostin has been detected in plasma^37,38^, our findings suggest its potential to serve as a diagnostic biomarker for rOC. In addition, the higher levels of *POSTN* expression, along with the phenotypes we identified that are associated with that higher level of expression, provide a foundation for future studies focused on targeted therapeutics for treatment of rOC.

Cancer stem cells (CSCs) are thought to be the source of recurrent disease^39^. Enrichment of CSCs and their role have been intensively studied in cancer, including ovarian cancer recurrence^40–42^. CSCs typically represent a small proportion of the total number of tumor cells, but they can drive both tumor development and progression. CSCs are not only responsible for primary tumor growth and metastasis, but also contribute to treatment failure, tumor progression, and relapse, since these cells are able to circumvent standard therapeutic approaches^39^. As the elimination of this cell population is critical for increasing treatment success, a deeper understanding of ovarian CSC pathobiology is needed, including their niche, signals for differentiation that trigger recurrence, including epithelial-mesenchymal transition (EMT)^43^, their signaling pathways, and how they interact with the tumor microenvironment. We found that a periostin-high environment increased cancer stemness in ovarian cancer, evidenced by an increase in the CSC side population (Fig. 4A) and by the expression of an established OC CSC marker, CD133 (Fig. 4B), providing a link between TME factors and CSC behavior. Targeting the factors that enhance CSC numbers, such as periostin in the TME, could provide a mechanism for reversing therapy resistance and reducing cancer recurrence.

We have shown that higher expression of periostin in cancer cell conditioned medium is associated with an increase in phospho-AKT levels, and that this is reduced by the addition of the AKT inhibitor MK-2206 (Fig. 6B). Thus, there appears to be a link between the phosphorylation of AKT and higher levels of exogenous periostin. AKT phosphorylation is frequently detected in ovarian cancer and can be targeted to disrupt ovarian tumor cell growth^44–47^. Activation of the AKT signaling pathway increases the expression of apoptosis inhibitor protein survivin, allowing for improved cancer cell survival^48^. Aberrant activity of AKT pathways in cancer is emerging as a focus for development of new cancer drugs^49^. Our data showing the connection between periostin-high expression in the TME in ovarian cancer and AKT activity support that targeting the AKT pathway could be a helpful strategy to treat OC patients with higher levels of periostin expression.

This is the first report showing that higher levels of periostin in preadipocyte cells is associated with an increased presence of lipids in the cytoplasm (Fig. 5A) and that this led to induction of the de novo fatty acid synthesis protein, FASN (Fig. 5B,C). This suggests the possibility that another potential role of periostin in the TME is to support production of a lipid-based energy source and possibly to regulate lipid metabolism in cancer cells. Indeed, using RNAseq analysis, we were able to see higher expression of *FASN* in cancer cells cultured in conditioned medium containing exogenous periostin. As a central regulator of lipid metabolism, FASN plays a critical role in the growth and survival of tumors with lipogenic phenotypes^50^. Due to the role of FASN in de novo fatty acid synthesis, our data suggest that lipid metabolism in cancer cells could be altered through increased *FASN* expression in response to an environment with higher periostin levels. Our data also provide evidence for communication between fatty acid metabolism outside in TME tissues and inside of cancer cells. A recent study showed that FASN inhibition increases mitochondrial priming and enhances breast cancer cell sensitivity to BCL2-targeting BH3 mimetics which may directly activate the apoptotic machinery and generate more potent and longer-lasting antitumor responses in a clinical setting^51^. We showed that OC cells are more resistant to the FASN inhibitor Cerulenin when they are cultured in the conditioned medium with higher periostin levels. Combined treatment with a FASN inhibitor and either carboplatin or paclitaxel showed a synergistic effect against OC cells grown conditioned media containing exogenous periostin. These findings together suggest that a critical integrated network between lipid metabolism in cancer cells and signals from the TME, including periostin, may provide new inroads for OC treatment.

Periostin is produced by adipocytes or fibroblasts and is secreted into the cancer extracellular matrix. We have shown that periostin can enhance cancer stemness. This, in turn, results in cancer cell chemoresistance, which may contribute to cancer recurrence. Additionally, FASN induced in cancer cells by elevated exogenous periostin plays important roles in lipid metabolism to generate excessive amounts of free fatty acids, which are then broken down into acetyl-CoA^52^. Acetyl-CoA then supports mitochondrial respiration through fatty acid oxidation to generate a greater amount of energy per unit mass compared to glucose^53^. This could at least partly, if not completely, support cancer cell regrowth or recurrence.

Previous studies suggested that periostin expression in OC cells promotes intraperitoneal tumor metastatic growth in immunodeficient mice^54^. Using a neutralizing antibody to periostin inhibited ovarian tumor growth and metastasis in a xenograft mouse model^55^. Due to the preferential expression of periostin in the stroma associated with cancer cells and the important connections between stromal periostin and patient prognosis^56^, we performed an in vivo study using CAOV2 OC cancer cells in the presence of CM^*POSTN*-high^. We found that tumors from OC cancer cells exposed to CM^*POSTN*-high^ were significantly larger than tumors from OC cells exposed to the control conditioned media.

Limitations of this study include that the tumor tissues used in the microarray analysis may have exhibited heterogeneity in tumor/stroma content, despite their selection and inclusion based on having ≥ 60% tumor content. A more comprehensive in vivo study of periostin function in recurrent cancer development is warranted in the future. In the current study, we used established ovarian cancer cell lines to study the role of periostin in epithelial ovarian cancer. These cell lines were established long ago and may not reflect the behavior of the original tumors from which they are derived. Patient-derived organoids or patient-derived xenograft models could be used in future studies to mimic the original characteristics of human cancers more closely.

In conclusion, we found that *POSTN* is frequently overexpressed in rOCs as compared with matched pOCs. We also showed enhanced cancer stemness and lipid production when periostin levels are elevated in the culture media, mimicking the TME. Our findings highlight the importance of periostin in the context of OC recurrence and may provide new insights relevant to biomarker development and therapeutic application.

## Materials and methods

All experiments were performed in accordance with relevant guidelines and regulations. All studies involving human research participants were performed in accordance with the Declaration of Helsinki (https://www.wma.net/policies-post/wma-declaration-of-helsinki-ethical-principles-for-medical-research-involving-human-subjects/).

### Tumor tissues

Primary and matched recurrent OC tissue sets (n = 16 sets) were from patients with stage III/IV high grade serous epithelial ovarian cancer. The primary tumor specimens were collected at the time of initial debulking surgery. Recurrent tumor samples were obtained from the same patients during “second-look” surgeries. Samples were obtained after patients provided written informed consent and were stored in the Duke Gynecologic Oncology Tissue Bank. This study was performed with Duke University Institutional Review Board approval, protocol Pro00027325. Clinical data for the paired pOC and rOC samples is provided in Supplementary Data Table 1.

### Microarrays

The tissues used for microarray data generation from pOCs and rOCs were selected if they exhibited more than 60% tumor content based on H&E staining from directly adjacent sections of the frozen tissues used for microarray analysis. RNA was extracted using the RNA Mini Kit according to the manufacturer’s protocol (Qiagen; Germantown, MD). Nucleic acid concentration and purity were assessed using a NanoDrop™ 2000 spectrophotometer (Thermo Fisher Scientific; Waltham, MA). RNA (1 µg) was analyzed for expression using the Affymetrix Human Genome U133 Plus 2.0 microarray, which includes 22,277 probes. The resulting gene expression data were normalized using the robust multiarray average algorithm (RMA)^57^. The Affymetrix gene expression data were analyzed by comparing values for primary and recurrent tumors using paired t test (alpha = 0.05).

### Immunohistochemistry

Frozen tumor tissues were cut into 5 μm sections using a microtome and the sections placed on slides. Immunohistochemistry staining for cytokeratin and periostin was carried out according to a previously published protocol^58^. Briefly, after blocking non-specific binding using blocking buffer, normal goat serum (Abcam, Ab7481), the slides were incubated at 4 °C overnight with a monoclonal antibody to human cytokeratin antigen (Abcam, Cat #ab53280, at 1:250), or monoclonal antibody to human periostin (R&D Systems, Cat # AF3548, at 10 µg/mL), or anti-human IgG (Abcam, Cat#109489, at 1:500). Immunodetection was carried out using the streptavidin–biotin-based Multi-Link Super Sensitive Detection System (4plus Universal HRP Detection System, Biocare Medical). Immunodetection was performed using a ZEISS light microscope. Micrographs were taken using brightfield imaging at 10X magnification with a 10X objective. Specimen areas were selected, and individual images were saved in a 24-bit RGB TIFF file format with a resolution of 1 μm/pixel using the ZEISS Microscopy Software. The slides with periostin staining were used for quantitative analysis using ImageJ (https://imagej.net/ij/)^59^. The images were converted 8-bit. The area of selection in square pixels (Area), average gray value within the selection (Mean), and the percentage of pixels within the selection (% Area) were measured and compared between primary and recurrent OC.

### Cell lines and cell culture

Mouse 3T3L1 fibroblast cells were purchased from ATCC (Cat # CL-173) and maintained in Dulbecco’s Modified Eagle Medium with high glucose (MilliporeSigma, Cat # D5796), 10% Bovine Calf Serum (MilliporeSigma, Cat # 12133C), and 1% Penicillin–Streptomycin (MilliporeSigma, Cat #p4333). HEK293T cells were purchased from ATCC (Cat # CRL-3216) and maintained in DMEM with high glucose, 10% Fetal Bovine Serum (FBS, ThermoFisher, Cat # 10082147), and 1% Penicillin–Streptomycin. Ovarian cancer cell lines used in this study are from the Duke Reproductive Sciences cell line repository and were originally obtained as gifts from Dr. Gordon Mills (HEYA8) and Dr. Jeff Boyd (CAOV2). SKOV3 cells were purchased from the ATCC (catalog ATCC HTB-77). HEYA8 and CAOV2 were reportedly originally derived from patients with high grade serous ovarian cancer (https://web.expasy.org/cellosaurus). CAOV2 was derived from ascites from a patient with HGSOC^60^. SKOV3 was derived from an ovarian serous cystadenocarcinoma (https://web.expasy.org/cellosaurus). A2780 cells were from an ovarian endometrioid adenocarcinoma (https://web.expasy.org/cellosaurus). All ovarian cancer cell lines were maintained in RPMI 1640 (ThermoFisher, Cat # A4192301) with 10% FBS and 1% Penicillin–Streptomycin. Cells were incubated at 37 °C in a humidified chamber with 5% CO_2_. The human cell lines undergo profiling to confirm genetic authenticity at the Duke University DNA Analysis Facility and are confirmed to be free of mycoplasma by the Duke Cell Culture Facility just prior to each expansion and preparation of frozen stocks.

### Generation of POSTN conditioned medium

3T3-L1 cells were stably transfected with a human *POSTN* lentiviral vector (pLenti-GIII-CMV-RFP-2A-Puro-*POSTN*, Applied Biological Materials Inc. Cat# LV268492) and control vehicle plasmid (pLenti-CMV-RFP-2A-Puro-Blank Vector, Applied Biological Materials Inc. Cat# LV591) to generate 3T3-L1 cell lines that do (3T3-*POSTN*) and do not (3T3-CTL) overexpress periostin. Lentivirus transfection was carried out according to the protocol described previously^61^. Due to the co-expression of red fluorescent protein (RFP) in cells, we were able to select *POSTN*-positive cells using flow-activated cell sorting. Overexpression of *POSTN* in 3T3-L1 cells (3T3-*POSTN*) as compared to 3T3-CTL cells was confirmed using RT-PCR with a human *POSTN*-specific probe as described below. Periostin protein expression in the medium was confirmed using an ELISA assay with an anti-periostin polyclonal antibody (R&D Systems, Cat # AF3548). 3T3-CTL cells were used to generate “control” conditioned medium (CM^*CTL*^), and 3T3-*POSTN* cells were used to generate conditioned medium with higher periostin levels (CM^*POSTNhigh*^). Media were collected for use after 48–72 h of culture and centrifuged at 1500 g for 5 min to remove cellular debris. These conditioned media (CM) were used for OC cell culture experiments as described and are referred to as CM^*CTL*^ or CM^*POSTNhigh*^.

### RT-PCR

Total RNA was extracted using RNA STAT-60 reagent according to the manufacturer’s instructions (AmsBio, Cat # CS-110). RT-PCR was carried out using 500 ng of total RNA in a 20 µL volume using SuperScript IV One-Step RT-PCR kit according to the manufacturer’s protocol (ThermoFisher, Cat# 12594025) with a Taqman probe specific to *POSTN* (Hs01566750, ThermoFisher, Cat# 4331182). Human Beta-2-Microglobulin (B2M) (ThermoFisher, Cat# 4333766T) served as an endogenous control for RNA input. The PCR reaction was performed at 95 °C for 10 min followed by 40 cycles of 95 °C for 15 s and 60 °C for 1 min. Relative RNA expression values were calculated using the threshold cycle (CT) values. All RT-PCR experiments were repeated three times, with 6 replicates each.

### ELISA

Enzyme-linked immunosorbent assays (ELISA) using the human periostin ELISA kit from Aviva Systems (Cat # OKCD09048) and the antibody included in the kit were performed according to the manufacturer’s instructions to compare periostin protein expression in the CM^*CTL*^ versus CM^*POSTNhigh*^. Three sets each of 3T3-CTL and 3T3-*POSTN* cells were cultured for 48–72 h followed by the collection of medium. After brief centrifugation to remove cellular debris, the CM was collected and used for ELISAs. Total protein concentration was determined using Pierce™ BCA Protein Assay Kit (ThermoFisher, Cat# 23225) according to the instructions from the manufacturer. Periostin protein expression from the ELISA assay was normalized with the total protein levels. A t-test was used to compare periostin expression from three replicates of 3T3-CTL and 3T3-*POSTN* samples. This test was repeated independently two times.

### Wound healing

OC cancer cells were cultured in 5 mL of either CM^*CTL*^ or CM^*POSTNhigh*^ in 6-well plates. Following incubation or transfection for 24 h, gaps were created in the cell monolayers using a sterile p200 pipet tip. Photomicrographs using brightfield imaging at 4X magnification with a 10X objective were taken at indicated time points using a Zeiss inverted microscope. Specimen areas were selected, and individual images were saved in a 24-bit RGB TIFF file format with a resolution of 1 μm/pixel using the ZEISS Microscopy Software. The width of the gap between the opposing edges was measured at four arbitrary points at each time point using ImageJ software (https://imagej.net/ij/)^59^. The means across all four measurements were calculated, normalized to the time 0-h, and compared between the CM^*CTL*^ and CM^*POSTNhigh*^ using two-way ANOVA.

### Invasion assay

We used a cell invasion assay kit (24-well; with a basement membrane) from Cell Biolabs (Cat # CBA-110). A HEYA8 OC cell suspension containing 150,000 cells was added to each insert of the invasion plate in 500 μL of either CM^*CTL*^ or CM^*POSTNhigh*^. Three repeats (3 wells) were included for each condition (CM^*CTL*^ or CM^*POSTNhigh*^). CM^*CTL*^ or CM^*POSTNhigh*^, each containing 20% FBS, was added to the lower chamber of the invasion plate. The cells were incubated for 48 h at 37 °C in a humidified chamber containing 5% CO_2_. Cells on the bottom of the invasion membrane were stained and quantified at OD 560 nm using a plate reader (BMG Labtech, POLARstar Omega) according to the protocol provided by the manufacturer (Cell Biolabs). This test was independently repeated three times.

### Chemosensitivity test

Since SKOV3, CAOV2 and HEYA8 cells exhibit chemoresistance to paclitaxel, diphtherotoxin, cisplatin, and doxorubicin^62^, A2780 cells were used for this test. A2780 cells were seeded into a 96-well plate (5,000 cells/well) in 100 μL of either CM^*CTL*^ or CM^*POSTNhigh*^ medium. Six replicates (6 wells) were performed for each CM and each treatment condition. After 24 h, carboplatin or paclitaxel was added at the indicated dose ranges. Following 72 h of drug treatment, cell viability was tested using the CellTiter-Glo® Luminescent Cell Viability Assay kit (Promega, Cat# G7570) according to the manufacturer’s instructions. The luminescence signal was recorded using a 96-well plate reader (BMG Labtech, POLARstar Omega). Readings were standardized to the mock treatment for each CM type and are reported as the percentage of viable cells relative to the mock treated cells. This test was independently repeated three times.

For chemosensitivity testing with cerulenin, HEYA8 cells were seeded into a 96-well plate at 4000 cells/well with 100 μL of CM^*CTL*^ or CM^*POSTNhigh*^. Six replicates per treatment condition and CM condition were performed. The cells were grown for 24 h followed by treatment for 72 h with either cerulenin at 10 μM, carboplatin at 3 μM, paclitaxel at 3 μM, 3 μM carboplatin + 10 μM cerulenin, or 3 μM paclitaxel + 10 μM cerulenin. At the end of the treatment, the CellTiter-Glo® Luminescent Cell Viability Assay kit reagent was added. Luminescence was measured using a 96-well plate reader. Readings were normalized to the control absorbance for each CM type, and relative to the mock treatment. This test was independently repeated three times.

### Flow cytometry for cell apoptosis

HEYA8 cells were cultured for 96 h with either CM^*CTL*^ or CM^*POSTNhigh*^ in triplicate for each CM condition. Apoptosis was induced for 24 h using paclitaxel at final concentrations of 100 nM and DMSO was used as a vehicle control. A similar experiment was performed using CAOV2 cells and Staurosporine at 1 μM to induce apoptosis for 18 h. After incubation, cells were collected and resuspended in PBS/1%FBS. After fixation using ice-cold 70% EtOH for two hours, cells were washed using PBS/1%FBS. Propidium iodide (PI) at 50 µg/mL and RNase A at 10 µg/mL were added to the cells. Apoptosis was analyzed using a Becton Dickinson FACS Vantage SE cell sorter and FACSDiva™ Software (BD Biosciences) using 488 nm excitation, and the emission was collected at 575 nm–610 nm. The cell cycle profile was obtained with the sub-G0/G1 (M1) peak representing the apoptotic population. This test was independently repeated three times.

### Cell apoptosis analysis by caspase-3 staining

HEYA8 cells were cultured for 96 h with either CM^*CTL*^, CM^*POSTNhigh*^, or CM^*POSTNhigh*^ + a neutralizing antibody for human periostin (R&D Systems, Cat # AF3548, at 10 µg/mL). Apoptosis was induced by treating cells with 100 nM paclitaxel for 24 h. Apoptosis was analyzed using the NucView® 488 caspase-3 assay kit (Biotium, Cat# 10402-T) which contains NucView® 488 Caspase-3 substrate. Counterstaining was done using the Hoechst 33342 DNA dye included in the kit. The staining was carried out according to the protocol provided by the manufacturer. Images were obtained using an AMG EVOS Imaging Microscope (AME-3206, AMG; Mill Creek, WA), at 20 × magnifications with a 10X objective. Specimen areas were selected, and individual images were saved in a 24-bit RGB TIFF file format at a resolution of 1280 × 960 pixels using the ImageScope software. This test was repeated twice. Caspase-3 staining intensity was measured using ImageJ.

### 3D cell culture

This experiment was carried out using HEYA8 cells since they form compacted spheroids under 3D cell culture conditions. OC cells were cultured in 5 mL of either CM^*CTL*^ or CM^*POSTNhigh*^ in ultralow attachment 6-well plates (MilliporeSigma, Cat # CLS3471). Spheroid formation was visualized using an inverted microscope (Zeiss) after 72 h of cell culture. Images were obtained using an inverted microscope (Zeiss) at 10X magnification and a 10X objective.

### Side population analysis

After 72 h of 3D culture in either CM^*CTL*^ or CM^*POSTNhigh*^, the side population of OC cells (HEYA8, CAOV2, and SKOV3) was measured using the flow cytometry approach^29,63,64^. Three wells were used for each CM condition. Briefly, after culture, the OC cells from each well were collected and stained for 90 min at 37 °C using 5 µg/mL Hoechst 33342 (bisBenzimide H 33342 trihydrochloride, MilliporeSigma, Cat# B2261). Verapamil (MilliporeSigma, Cat# V4629), an inhibitor of ABC transporters, was used as a negative control and was added to a final concentration of 50 μM along with the Hoechst 33342 dye. Before FACS analysis, propidium iodide (PI) solution was added to a final concentration of 1 μg/mL to identify nonviable cells. FACS analysis was carried out with a dual laser flow cytometer (Becton Dickinson FACS Vantage SE cell sorter) and FACSDiva™ Software (BD Biosciences). Hoechst dye was excited at 355 nm by an ultraviolet laser, and the emission was measured at two wavelengths using a 424/44 (Hoechst blue) and a 585/42 (Hoechst red) band-pass filters. PI excitation was attained using a 488 nm laser and detected after passing through a 630/22 band-pass filter. PI-positive dead cells and debris were excluded. The side population from cancer cells cultured with CM^*CTL*^ or CM^*POSTNhigh*^ was compared using a paired student t-test. The experiments were performed in duplicate for each OC cell line.

### Flow cytometry

CAOV2 cells were cultured with either CM^*CTL*^ or CM^*POSTNhigh*^ for 96 h. The cell pellets were collected by centrifugation and resuspended in 50 μL of FcR Blocking Reagent (Miltenyi Biotec, Cat#130-090-901) containing 0.5% bovine serum albumin (Sigma, St Louis, Cat# A9576) and 0.05% sodium azide in phosphate-buffered saline. Mouse anti-human CD133/1-PE monoclonal antibodies (Miltenyi Biotec, Cat#130-080-801) were then added at a 1:11 dilution and incubated for 30 min at 4 °C in the dark. Isotype controls were also used, including mouse IgG1-PE (Miltenyi Biotec, Cat# 130-092-212). After two cycles of washing with PBS, the cells were fixed using 4% paraformaldehyde (PFA) for 15 min at room temperature. The supernatant was removed, and the cells were resuspended in 500 μL of PBS and transferred through a 35-nm nylon mesh into polystyrene tubes and stored at 4 °C in the dark until processing for flow cytometry analysis. Flow cytometric data were obtained on a BD FACSCanto-II instrument (BD Biosciences) at the Duke Comprehensive Cancer Center Flow Cytometry Shared Resource Facility. The data was analyzed and compared between CAOV2 cells cultured with CM^*CTL*^ or CM^*POSTNhigh*^ for their CD133-positive populations. The test was performed in triplicate for each condition.

### 3T3-L1 differentiation and oil red O assay

To induce 3T3L1-CTL and 3T3L1-*POSTN* cells to differentiate into adipocyte-like cells, the cells were treated using the reagents from the 3T3-L1 differentiation kit according to instructions (MilliporeSigma, Cat #DIF001). Briefly, the cells were cultured until confluent with preadipocyte medium (DMEM medium with 10% bovine calf serum, 100 units/mL penicillin, and 100 μg/mL streptomycin). The preadipocyte medium was then replaced with differentiation medium containing 1 μL/mL of Differentiation Cocktail in DMEM/F12 (1:1) medium with 10% FBS. Three days after the differentiation, the medium was replaced with 1 μL/mL of insulin containing DMEM/F12 (1:1) with 10% FBS. Lipid droplet formation and accumulation was visible by light microscopy about 7–10 days after addition of the differentiation medium. The cells were fixed with 10% formalin and stained with Oil Red O (MilliporeSigma, Cat #MAK194) nine days after differentiation initiation. Micrographs were taken using an inverted microscope (Zeiss) at 10X magnification and a 10X objective. Specimen areas were selected, and individual images were saved in a 24-bit RGB TIFF file format with a resolution of 1 μm/pixel using the ImageScope software. This test was independently repeated three times.

### RNAseq

Due to their low endogenous expression of *POSTN* (based on microarray gene expression data for OC cell lines), HEYA8 cells were used for this study. HEYA8 cells were cultured in either CM^*CTL*^ or CM^*POSTNhigh*^ for 72 h under 3D cell culture conditions. RNA was extracted using RNA STAT-60 (Amsbio, Cat#CS-111). Six replicates of each (CM^*CTL*^ or CM^*POSTNhigh*^) were used for generation of the RNA sequencing data. We confirmed the increase in cell proliferation and invasion and apoptosis inhibition in the cells cultured in CM^*POSTNhigh*^ before submitting 1 μg of RNA to the Genomic Analysis and Bioinformatics Core at Duke University for generation of RNA Seq data using the NovaSeq 6000 system. The Illumina Truseq mRNA stranded RNA-Seq Library Prep Kit protocol was used for sequencing library construction. Libraries were checked for quality and quantified using the Bioanalyzer 2100 (Agilent, Santa Clara, CA, USA), before being sequenced on one S1 lane of an Illumina NovaSeq 6000 instrument using 150 base paired-end sequencing. The quality of the sequencing output was assessed using FastQC v.0.11.9 (http://www.bioinformatics.babraham.ac.uk/projects/fastqc/). RNA-seq data was processed using the fastp toolkit1 to trim low-quality bases and Illumina sequencing adapters from the 3’ end of the reads. Only reads that were 20nt or longer after trimming were kept for further analysis. Reads were mapped to the GRCh38v93 version of the human genome and transcriptome^65^ using the STAR RNA-seq alignment tool^66^. Reads were kept for subsequent analysis if they mapped to a single genomic location. Gene counts were compiled using the featureCounts tool^67^. Only genes that had at least 10 reads in any given library were used in subsequent analysis. Normalization and differential expression were carried out using the DESeq2 Bioconductor^68^ package with the R statistical programming environment. The false discovery rate was calculated to control for multiple hypothesis testing. Gene set enrichment analysis^69^ was performed to identify gene ontology terms and pathways associated with altered gene expression for each of the comparisons performed. The RNAseq dataset is available at https://doi.org/10.7924/r4qf8wr73.

### Western blotting

HEYA8 cells were seeded at equal cell numbers (1 × 10^6^) onto 100 mm ultralow attachment Petri dishes for 3D culture as described above. Each cell line was plated in four conditions: CM^*CTL*^, CM^*POSTNhigh*^, CM^*CTL*^ + 10 μL MK-2206 2HCl (a selective AKT inhibitor from Selleckchem, Cat#S1078), and CM^*POSTNhigh*^ + 10 μL MK-2206 2HCl. The Evos FL Cell Imaging system was used to take micrographs of 3D cell cultures every 24 h to check spheroid formation. Cells were harvested after 72 h of culture for protein analysis with western blotting. The cell pellets were incubated in NP-40 cell lysis buffer (ThermoFisher, Cat#85124) for 30 min on ice. Cell lysates (50 µg) were loaded into 4–20% PROTEAN® TGX™ Precast Protein Gels (BioRad, Cat# 5678081). After overnight transfer to nitrocellulose membranes, the blot was incubated with primary antibodies, including anti-phospho-AKT (1:2500, Cell Signaling, Cat# #9271), anti-AKT (1:1000, Cell Signaling, Cat#9272), and anti-GADPH (1:2000, ThermoFisher, Cat# PA1-987). After secondary antibody incubation, ECL Western Blot Substrate kit (Bio-Rad, Cat# 1705060S) was used to detect the signals, which were imaged using the Chemidoc imaging system from Bio-Rad. The blots for p-AKT, AKT and GAPDH were cropped from the full images, which are provided in Supplementary Fig. 5.

### Ovarian cancer xenograft mouse model

The experimental animal protocol for this study was approved by the Duke Institutional Animal Care and Use Committee (IACUC protocol number A223-21-11). All methods are reported in accordance with ARRIVE guidelines. All experiments were performed in accordance with the approved IACUC protocol including animal number, cancer cell injections, surgical procedures to remove tumors, and euthanasia. The study plan was created using the Experimental Design Assistant showing a simple comparative study for the effect of periostin on cancer growth of two different groups, with CM^*POSTN*high^ or CM^CTL^ medium-cultured CAOV2 cells (also see below, “Statistical analysis” section). The corresponding author (ZH) was the only person aware of the treatment group allocation. Eighteen six-week-old female athymic nude mice were purchased from Charles River Laboratories. Only female mice were included as ovarian cancer only affects females. Mice were maintained on a 12:12 h light:dark schedule and were fed ad libitum with standard mouse chow and had free access to fresh water. Mice were housed in micro isolator caging at 4–5 animals per cage and were maintained on corn cob bedding material. Room temperature was maintained within the thermoneutral zone and humidity within limits recommended in the NIH Guide for the Care and Use of Laboratory Animals^70^. Caging was changed once a week. Environmental enrichment was provided by the Division of Laboratory Animal Resources staff. CAOV2 cells were prepared either in CM^*POSTNhigh*^ or CM^*CTL*^ medium and then mixed at a 1:1 ratio with Matrigel Growth Factor Reduced (GFR) Basement Membrane Matrix (Corning, Cat#354230). The CAOV2 cell line was used to generate the xenograft model because of its high-grade serous OC source and established tumorigenicity after subcutaneous (SubQ) or intraperitoneal (IP) cell injection^19,33^. Eighteen mice were randomly assigned into two study groups. Ten mice were injected subcutaneously in the upper right flank with 5 × 10^6^ cells each of CAOV2 grown with CM^*POSTNhigh*^ (CM^*POSTNhigh*^ group). Eight mice were used as controls and were injected in the same manner with 5 × 10^6^ cells each of CAOV2 grown in CM^*CTL*^ mixed with GFR Matrigel at a 1:1 ratio (CM^*CTL*^ group). Tumors were surgically removed 20 days after cancer cell injection. Both buprenorphine SR-LAB and isoflurane inhalation were used for anesthesia for mouse surgery. Buprenorphine SR-LAB was administered 30 min before surgery, at 1.0 mg/kg by subcutaneous (SC) injection. Isoflurane (2% in 6 L/min oxygen) was administered throughout the surgical procedure and eye ointment was used to prevent drying. After betadine and alcohol disinfection (repeated 3 times), a 1 cm incision was made through the skin at the tumor site using a sterile scalpel. All visible tumor tissues were removed using surgical scissors. Surgical staples were used to close the incision. Buprenorphine SR-LAB was used immediately after surgery at 1.0 mg/kg SC. The mice were returned to their cages after awakening. Mice were monitored daily following surgery to assess healing, any surgical site bleeding, and overall health. Tumor length and width were measured using calipers, and then the tumors were frozen at − 80 °C. Tumor volume was calculated using the formula $V=\frac{{W}^{2} \times L}{2}$^71^. At the end of the study, or when the animals reached their humane endpoints, euthanasia was performed using CO_2_ inhalation with decapitation as the secondary means to confirm death. This method is consistent with the recommendations of the Panel on Euthanasia of the American Veterinary Medical Association and authorized by Duke IACUC (Institutional Animal Care and Use Committee). Duke’s Division of Laboratory Animal Resources veterinary support staff were responsible for the anesthesia and surgical procedures. Measurement and dissection of tumors were performed by research assistants.

### Statistical analysis

Chemosensitivity assays were evaluated using a one-way ANOVA test with replication. The statistical means across all measurements from wound healing gaps were calculated and compared between the CM^*CTL*^ and CM^*POSTNhigh*^ using two-way ANOVA using Prism 9 (GraphPad Software, LLC). The comparison of *POSTN* expression or normalized *POSTN* expression between pOC and rOC was performed using paired t test using Prism 9 (GraphPad Software, LLC). Gene expression from qRT-PCR was analyzed and compared between groups with a paired student t test using Prism 9 (GraphPad Software, LLC). In the mouse tumor study, the mean tumor volumes were compared between the CM^*CTL*^ and CM^*POSTNhigh*^ groups using the Mann Whitney U test. For all statistical analysis, p values < 0.05 were considered statistically significant.

### Supplementary Information

Supplementary Figures.

Supplementary Tables.

## Data Availability

The data that support the findings of this study are available on request from the corresponding author. RNAseq data generated from HEYA8 cells cultured in high periostin media is available at https://doi.org/10.7924/r4qf8wr73. The Affymetrix Human Genome U133 Plus 2 Array data from 16 primary and recurrent serous epithelial ovarian cancers is available from the Duke Research Data Repository (2021). 10.7924/r43f4sx2k.
