# Supplementary material for: Periostin facilitates ovarian cancer recurrence by enhancing cancer stemness
Source: Sci Rep. 2023 Dec 4;13:21382. doi: 10.1038/s41598-023-48485-8 (PMC10695946; doi:10.1038/s41598-023-48485-8)
Supplement: Supplementary file 2 — Supplementary Tables. [file 41598_2023_48485_MOESM2_ESM.docx]

***Supplementary Table 1:* Clinical Data, Matched Primary and Recurrent Ovarian Cancers**

| **Tumor ID**  **(pOC/rOC)** | **Age at Diagnosis**  **(year)** | **Final Pathology FIGO Grade** | **CA125 at End of Primary Therapy** | **Platinum**  **Sensitivity/Resistance**  **(pOC/rOC)** | **Months to Recurrence Following Surgery** | **Survival (Months)** |
| --- | --- | --- | --- | --- | --- | --- |
| 1P/1R | 35 | 2 | >10 | S/S | >6 | >36 |
| 2P/2R | 54 | 1 | >10 | R/R | >6 | >36 |
| 3P/3R | 57 | 3 | Not detected | S/S | >6 | <36 |
| 4P/4R | 66 | 3 | >10 | R/R | >6 | >36 |
| 5P/5R | 50 | 2 | ≤10 | R/R | >6 | <36 |
| 6P/6R | 72 | 3 | ≤10 | R/Unknown (Pt. died of disease) | >6 | >36 |
| 7P/7R | 59 | 2 | ≤10 | S/S | >6 | <36 |
| 8P/8R | 46 | 2 | Unknown | R/S | ≤6 | <36 |
| 9P/9R | 57 | 3 | >10 | R/R | ≤6 | <36 |
| 10P/10R | 62 | 2 | >10 | R/R | >6 | <36 |
| 11P/11R | 61 | 3 | >10 | S/R | >6 | >36 |
| 12P/12R | 46 | 2 | >10 | R/S | >6 | >36 |
| 13p/13R | 70 | 3 | ≤10 | S/R | >6 | <36 |
| 14P/14R | 57 | 2 | Unknown | Unknown/Unknown | >6 | <36 |
| 15P/15R | 58 | 2 | Unknown | Unknown/Unknown | >6 | >36 |
| 16P/16R | 69 | 2 | Not detected | S/S | >6 | >36 |

***Supplementary Table 2:* Measurements of POSTN Positive Staining Using ImageJ for POSTN IHC Staining in Figure 1C.**

|  | **Area^1^** | **Mean^2^** | **% Area^3^** |
| --- | --- | --- | --- |
| **Primary OC** | 713,942 | 20.2 | 7.9 |
| **Recurrent OC** | 342,485 | 87.9 | 34.5 |
| 1. Area of selection in square pixels  2. Mean gray value; the sum of the gray values of all pixels in the selected area divided by the total number of pixels  3. Area fraction; the percentage of pixels in the selection that have been highlighted | | | |

***Supplementary Table 3:* Measurements of Caspase-3 Positive Staining in Figure 3C Using ImageJ.**

|  | **Area^1^** | **Mean^2^** | **% Area^3^** |
| --- | --- | --- | --- |
| **CM*^CTL^*** | 26,180 | 178.4 | 69.9 |
| **CM*^POSTNhigh^*** | 26,180 | 88.7 | 34.8 |
| **CM*^POSTNhigh^*** **+ POSTN antibody** | 26,180 | 168.7 | 66.2 |
| 1. Area of selection in square pixels  2. Mean gray value; the sum of the gray values of all pixels in the selected area divided by the total number of pixels  3. Area fraction; the percentage of pixels in the selection that have been highlighted | | | |

***Supplementary Table 4:* The area and dimensions of spheroids in Supplementary Figure 6 measured by ImageJ.**

| **Spheroid** | **Area^1^** | **Dimensions^2^** |
| --- | --- | --- |
| **CM*^CTL^*** | 8,532.9 | 91.7 x 109.9 |
| **CM*^POSTNhigh^*** | 20,696.0 | 217.7 x 74.1 |
| **CM*^CTL^* + MK2206** | 2,096.2 | 57.9 x 36.4 |
| **CM*^POSTNhigh^* + MK2206** | 2,670.2 | 61.5 x 29.0 |
| 1. Area - Area of selection in square micrometers.  2. Dimensions - length x width, in square micrometers. | | |

***Supplementary Table 5:* Tumor volumes measured (mm^3^) on Day 11 and Day 18 post-tumor cell injection.**

|  |  | **Mouse**  **1** | **Mouse**  **2** | **Mouse**  **3** | **Mouse**  **4** | **Mouse**  **5** | **Mouse**  **6** | **Mouse**  **7** | **Mouse**  **8** | **Mouse**  **9** | **Mouse**  **10** |
| --- | --- | --- | --- | --- | --- | --- | --- | --- | --- | --- | --- |
| **Day11** | **CM*^CTL^*** | 0 | 33.0 | 0 | 16.9 | 0 | 0 | 27.9 | 18.0 | X | X |
|  | **CM*^POSTNhigh^*** | 39.5 | 2.3 | 33.0 | 44.5 | 0 | 19.6 | 31.0 | 0 | 0 | 0 |
| **Day18** | **CM*^CTL^*** | 0 | 116.6 | 0 | 255.9 | 0 | 297.5 | 87.1 | 108.0 | X | X |
|  | **CM*^POSTNhigh^*** | 122.8 | 72.0 | 194.2 | 352.0 | 228.1 | 121.8 | 90.8 | 179.6 | 59.2 | 0 |
| 0 – No tumor detected  X – No mice | | | | | | | | | | | |
