# Supplementary material for: Periostin facilitates ovarian cancer recurrence by enhancing cancer stemness
Source: Sci Rep. 2023 Dec 4;13:21382. doi: 10.1038/s41598-023-48485-8 (PMC10695946; doi:10.1038/s41598-023-48485-8)
Supplement: Supplementary file 1 — Supplementary Figures. [file 41598_2023_48485_MOESM1_ESM.docx]

***Supplementary Figure 1***

***Supplementary Figure 2***

***Supplementary Figure 3***

**A.**

**B.**

***Supplementary Figure 4***

**A.**

**B.**

***Supplementary Figure 5***

***Supplementary Figure 6***

***Supplementary Figure 1*.** *POSTN* expression was normalized using the stromal specific genes, *MTSS1* and *RAPGEF2*. Normalized *POSTN* expression in primary OC and recurrent OC were compared (p=0.008 for *MTSS1* normalization, and p=0.006 for *RAPGEF2* normalization).

***Supplementary Figure 2.*** *POSTN* RNA expression by RT-PCR in 3T3 cells stably transfected with a *POSTN* expression vector (3T3-POSTN) relative to cells with the blank vector (3T3-CTL), normalized with the internal control *B2*M (p=0.003). Results shown for three experiments, each performed in duplicate.

***Supplementary Figure 3*.** **Increased resistance to apoptosis with high levels of periostin in the microenvironment in CAOV2 cells.** **A.** CAOV2 cells were cultured with either CM*^POSTNhigh^* (lower panel) or CM*^CTL^* (upper panel). CAOV2 cells exhibit a reduced number of cells undergoing apoptosis induced by staurosporine (1µM) for 18 hours when cultured under CM*^POSTNhigh^* compared to cells grown under CM*^CTL^*. **B.** Summary graph for comparison of apoptosis percentages in CAOV2 cells from two independent tests.

***Supplementary Figure 4.* CM*^POSTNhigh^* culture enhances cancer cell stemness.** **A.** The CAOV2 and SKOV3 cancer stem cell-like side population (SP) increases in Ucells grown under CM*^POSTNhigh^* compared to cells grown under CM*^CTL^* following H33342 fluorescence staining, using verapamil treatment as a control. **B.** Average percentage of SP cells for CAOV2 (left panel, from three tests) and SKOV3 (right panel, from two tests).

***Supplementary Figure 5.*** Original Western blots gel/blot images for p-AKT (A), GAPDH (B), and total AKT (C). Each panel (A, B and C) has the same lane order as the same blot was consecutively stripped and reprobed: Lane 1: HEYA8 cells, CM*^CTL^*; Lane 2: HEYA8 cells, CM*^POSTNhigh^*; Lane 3: HEYA8 cells, CM*^CTL^* and MK2206; Lane 4: HEYA8 cells, CM*^POSTNhigh^* and MK2206; Lane 5: CAOV2 cells, CM*^CTL^*; Lane 6: CAOV2 cells, CM*^POSTNhigh^*; Lane 7: CAOV2 cells, CM*^CTL^* and MK2206; Lane 8: CAOV2 cells, CM*^POSTNhigh^* and MK2206.

***Supplementary Figure 6.*** Spheroids shown in Figure 6 were outlined and the area and dimensions were measured using ImageJ.
